# Effect of *Salmonella enterica* serovar Typhimurium VNP20009 and VNP20009 with restored chemotaxis on 4T1 mouse mammary carcinoma progression

**DOI:** 10.18632/oncotarget.16830

**Published:** 2017-04-04

**Authors:** Sheryl L. Coutermarsh-Ott, Katherine M. Broadway, Birgit E. Scharf, Irving C. Allen

**Affiliations:** ^1^ Virginia Tech, VA-MD College of Veterinary Medicine, Department of Biomedical Sciences and Pathobiology, Blacksburg, VA, USA; ^2^ Virginia Tech, Department of Biological Sciences, Blacksburg, VA, USA

**Keywords:** bacterial therapeutic, breast cancer, chemotaxis, CheY, metastasis

## Abstract

A variety of bacterial strains have been evaluated as bio-therapeutic and immunomodulatory agents to treat cancer. One such strain, *Salmonella enterica* serovar Typhimurium VNP20009, which is attenuated by a purine auxotrophic mutation and modified lipid A, is characterized in previous models as a safely administered, tumor colonizing agent. However, earlier work tended to use less aggressive cancer cell lines and immunocompromised animal models. Here, we investigated the safety and efficacy of VNP20009 in a highly malignant murine model of human breast cancer. Additionally, as VNP20009 has recently been found to have a defective chemotaxis system, we tested whether restoring chemotaxis would improve anti-cancer properties in this model system. Exposure to VNP20009 had no significant effect on primary mammary tumor size or pulmonary metastasis, and the tumor colonizing process appeared chemotaxis independent. Moreover, tumor-bearing mice exposed to *Salmonella* exhibited increased morbidity that was associated with significant liver disease. Our results suggest that VNP20009 may not be safe or efficacious when used in aggressive, metastatic breast cancer models utilizing immunocompetent animals.

## INTRODUCTION

The use of bacteria as bio-therapeutic agents for cancer treatment has a long and interesting history. Some of the earliest documented, large scale observations supporting the use of bacteria as bio-therapy came from William B. Coley over 150 years ago, who reported that a significant fraction of cancer patients went into remission or were completely cured of their cancer following the development of post-operative bacterial infections [1]. Indeed, early studies between 1868 and 1944 evaluated the direct injection of bacterial toxins and bacterial broth cultures to treat malignant tumors, with ample cases reported of both successes and failures [2–4]. Over the last few decades, numerous studies have emerged that re-invigorated the field of bacteria-based bio-therapeutics for the treatment of cancer. Indeed, several bacterial genera have been evaluated in pre-clinical cancer models, including *Bifidobacterium*, *Clostridium* and *Salmonella enterica* serovar Typhimurium [5–7]. However, as evidenced by the volume of studies in the current literature, *Salmonella* is by far the most extensively evaluated and characterized bacterial genus currently being explored as a cancer bio-therapeutic agent [8].

*Salmonella* is an attractive model for studying tumor targeting due to its facultative anaerobic nature, allowing for the oxygenated circumference of the tumor as well as the hypoxic core region to be colonized by bacteria (reviewed in [9]). In addition, *Salmonella* can be manipulated genetically with relative ease and possesses a facultative intracellular lifestyle [10]. Importantly, attenuated *S*. Typhimurium has resulted in decreased tumor growth in mice bearing B16F10 melanoma and has been suggested to be able to colonize solid tumors up to a reported 9,000 times greater than the liver [11]. This is significant, as it reflects the specificity of the bacteria to colonizing cancerous tissue as opposed to clearance from the host.

Several strains of *S*. Typhimurium have been investigated for the purpose of tumor targeting and chemotherapy delivery, including VNP20009, A1-R, and CRC2631 ([12–14], reviewed in [15]). VNP20009 was constructed by Low et al. from strain 14028 through selection of hyperinvasion by chemical and UV mutagenesis, targeted deletions resulting in purine auxotrophy, and attenuation by modification of lipid A [16]. Success of the strain, due to its anticancer effects and high safety profile in pre-clinical animal models, resulted in a Phase 1 Clinical Trial in 2001. In this study, VNP20009 was introduced as a treatment to patients with nonresponsive metastatic melanoma or renal cell carcinoma. Although colonization was observed for some patients, treatment with VNP20009 did not result in tumor regression [17]. However, attempts to maximize bacterial tumor colonization and anticancer effects continue to be investigated.

Many virulent properties of *S*. Typhimurium have been evaluated for optimization of bacterial localization and retardation of tumors. These include components of virulence such as pathogenicity islands SPI-1 and SPI-2, motility, chemotaxis, biofilm formation and metabolism ([18, 19], reviewed in [20]). Utilization of chemotaxis is a particularly interesting concept, because the machinery can be manipulated to facilitate bacterial colonization of specified regions of tumors based on the nutrient content. Generally, it has been found that bacterial chemotaxis is favorable for tumor spheroid colonization *in vitro*, with specific receptors facilitating tumor microenvironment localization [21, 22]. In contrast, the role of chemotaxis *in vivo* is controversial, reasons for which may include the use of different bacterial strains, cancer cell lines, and experimental conditions. In a CT26 colon carcinoma model, intravenously injected *S*. Typhimurium SL7207 with a functional chemotaxis system yielded an advantage to tumor colonization over chemotaxis deficient strains 12 hours after injection, but no differences were observed after 24 hours [18]. A few studies have assessed bacterial chemotaxis in an *in vivo* 4T1 mammary carcinoma model. Using high-throughput screening of single-gene deletion mutants of *S*. Typhimurium 14028, chemotaxis was found to be beneficial for tumor colonization 2 days after infection [8]. However, chemotaxis had no significant influence after 2 days for strain SL1344 [23]. VNP20009 was recently discovered to be deficient in chemotaxis, due to a non-synonymous SNP in the gene encoding the chemotaxis two component response regulator, *cheY* [24]. Upon replacing the deficient copy of *cheY* with the wild-type copy, chemotaxis was recovered to 70% of the parental strain [24].

In the present study, we sought to evaluate the effects of VNP20009 and VNP20009 with restored chemotaxis in the context of 4T1 mammary carcinoma progression. The 4T1 cell line in mice is an attractive model of triple-negative breast cancer due to its high degree of clinical relevance, the ability to use immunocompetent mice, and the fact that metastasis occurs through a highly predictable mechanism that accurately mimics human breast cancer malignancies [25]. Previous studies have evaluated VNP20009 in other models of tumorigenesis, including diverse sub-types of cancer in mouse models, prostate, pancreatic, and breast cancer, as well as sarcoma and glioma [13, 26–30]. However, the majority of these prior reports were focused on xenograft studies using immunocompromised animals inoculated with minimally aggressive tumors and nominal assessments of either metastasis or systemic pathology. Here, we provide a robust evaluation of 4T1 cancer progression in the presence of chemotaxis positive or chemotaxis deficient VNP20009 as indicated by morbidity and mortality evaluations of wild-type BALB/c mice, assessments of primary tumor burden, measurements of metastatic cell potential in the lungs, immune system function, and comprehensive pathological evaluations over the time course of the experiment. Our results reveal that treatment with VNP20009 does not have a significant effect on primary mammary tumor growth or pulmonary metastasis. While we did observe tumor colonization, this process appeared to be independent of chemotaxis. Furthermore, tumor bearing mice infected with VNP20009 demonstrated increased morbidity that was associated with significant liver disease. Together, these data suggest that VNP20009 may not be safe or efficacious in models of highly aggressive, metastatic cancer.

## RESULTS

### *S*. Typhimurium infection in combination with the presence of mammary tumors increased morbidity

**Figure 1:**
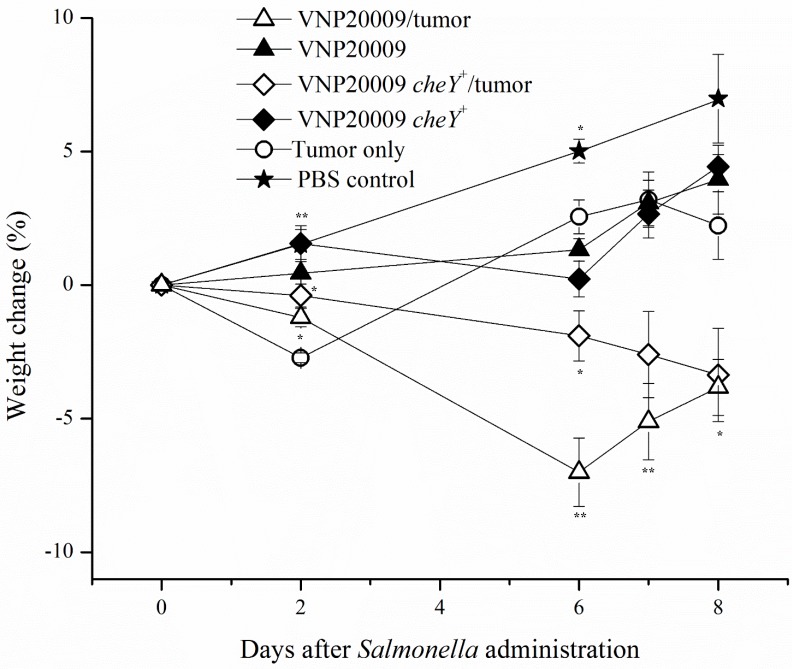
Weight change following exposure to *Salmonella* 4T1-tumor bearing animals or non-tumor bearing control animals were injected intravenously with *S*. Typhimurium VNP20009, *S*. Typhimurium VNP20009 *cheY*^+^, or PBS. Animals were monitored three times per week and weights were measured twice weekly. PBS control, *n* = 3; *S*. Typhimurium only, *n* = 4; Tumor only, *n* = 3; VNP20009 and tumor, *n* = 8*;* VNP20009 *cheY*^+^ and tumor, *n* = 8. Data points and error bars denote mean ± SEM. Statistical significance was determined in relation to the tumor only sample by a two-tailed Student's *T-test* (*p < 0.05, **p < 0.01).

It is well known that multiple strains of bacteria can colonize a variety of *in vivo* tumors. *S*. Typhimurium VNP20009 has been previously reported to exhibit preferential tumor colonization and dosage-related safety in a variety of tumor-infected animals including monkeys, pigs, and mice, as well as, humans [12, 17, 31]. The recently constructed VNP20009 derivative with restored bacterial chemotaxis, VNP20009 *cheY*^+^, has yet to be assessed *in vivo* for tumor colonizing ability [24]. We therefore evaluated the effects of intravenously (i.v.) injected *S*. Typhimurium VNP20009 on primary tumor burden and pulmonary metastasis in the 4T1 mammary carcinoma model over the course of 8 days. Mice were injected with 1.2 × 10^6^ 4T1 mammary carcinoma cells and tumors were allowed to grow for 16 days. Animals were then i.v. injected with 2 × 10^4^ CFUs of either *S*. Typhimurium VNP20009, which is non-chemotactic, or a chemotaxis-restored derivative VNP20009 *cheY*^+^. Control animals were injected i.v. with the same volume of PBS. Our results showed that animals with both tumors and either VNP20009 strain had a markedly increased morbidity when compared to all other treatment groups (Figure 1). Animals that received both tumors and VNP20009 had an 8–10% decrease in weight compared to either VNP20009 strain or 4T1 groups alone, suggesting a synergistic effect between the tumor and bacterial infection (Figure 1). The effect was independent of the chemotactic ability of the VNP20009 strains.

### Systemic exposure to *S*. Typhimurium had no effect on primary tumor size

**Figure 2:**
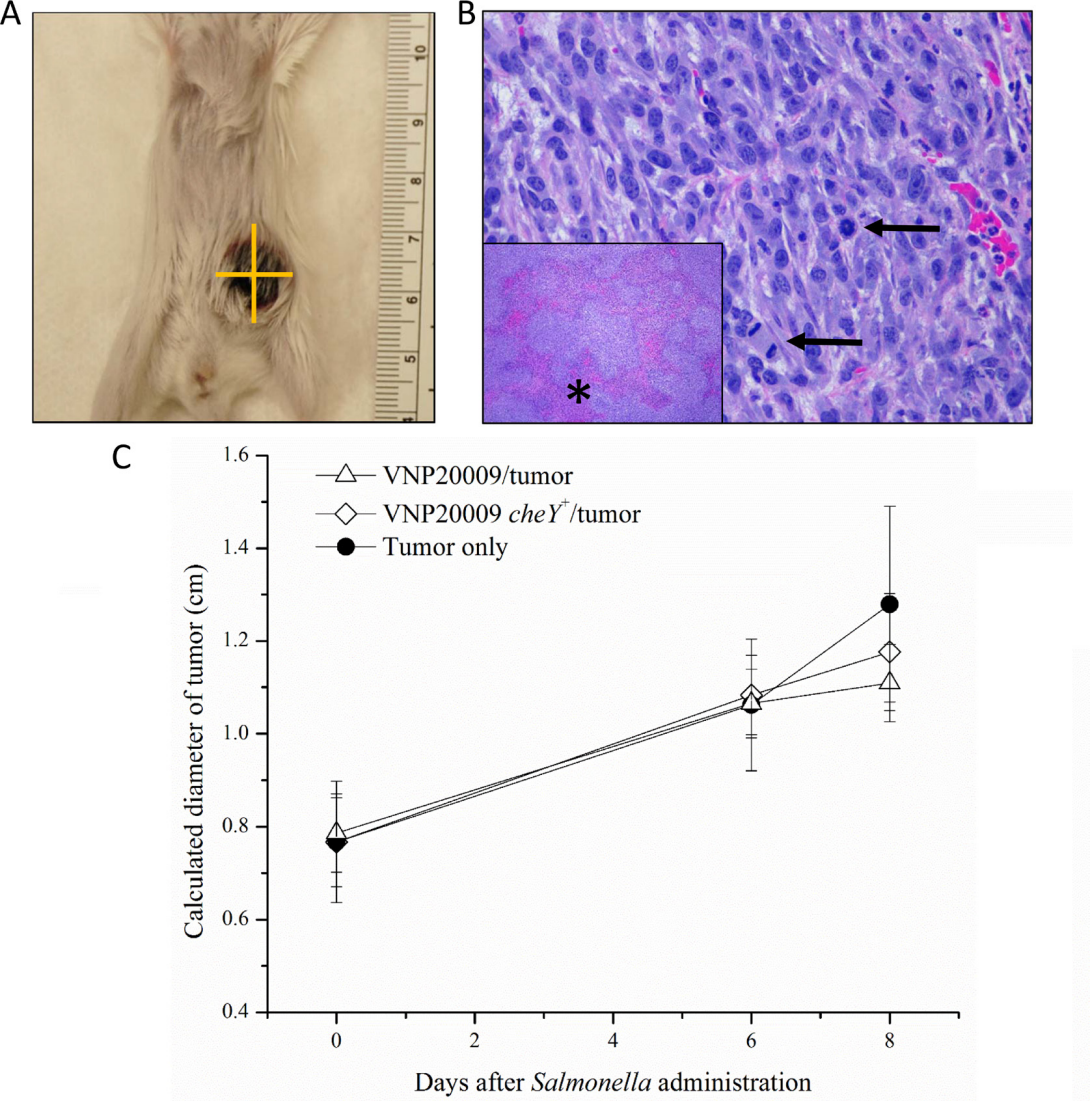
Mammary tumors developed in all 4T1-injected animals (**A**) Grossly, tumors appeared as spherical, raised, firm masses with an ulcerated surface. (**B**) Histologically, tumors were composed of a pleomorphic population of neoplastic epithelial cells with numerous mitotic figures (arrows) and large areas of necrosis (inset, asterisk). (**C**) All animals injected with 4T1 cells developed mammary tumors. Tumors were monitored three times per week and measurements were recorded twice per week. Measurements were taken using calipers and measuring two perpendicular diameters. The square root of the product of these two measurements was taken to give a calculated diameter for each tumor. PBS control, *n* = 3; *S*. Typhimurium only, *n* = 4; Tumor only, *n* = 3; VNP20009 and tumor, *n* = 8; VNP20009 *cheY*^+^ and tumor, *n* = 8. Data points and error bars denote mean ± SEM.

Because multiple studies suggested the importance of *S*. Typhimurium chemotaxis on both the ability of bacteria to colonize tumor tissue and to influence tumor growth, we investigated the effects of VNP20009 and VNP20009 *cheY*^+^ on primary mammary tumor growth in the 4T1 model. Mice were monitored three times per week and tumor size was recorded twice per week (Figure 2A). All animals injected with 4T1 cells developed mammary tumors that progressed as expected [25]. Control animals receiving PBS injections into the mammary fat pad instead of 4T1 cells did not develop spontaneous mammary tumors. Grossly, tumors appeared as spherical, raised, firm masses with an ulcerated surface (Figure 2A). Histologically, mammary tumors from all tumor-bearing animals appeared similar. All of the mammary tumors were composed of typical neoplastic epithelial cells with marked atypia and numerous mitotic figures (Figure 2B). Large areas of necrosis were also present (Figure 2B). There was no statistically significant difference in the size of the primary tumor between animals infected with either VNP20009 strain or those only bearing 4T1 mammary tumors (Figure 2C).

### Systemic exposure to *S*. Typhimurium had no effect on the presence or amount of pulmonary metastasis

**Figure 3:**
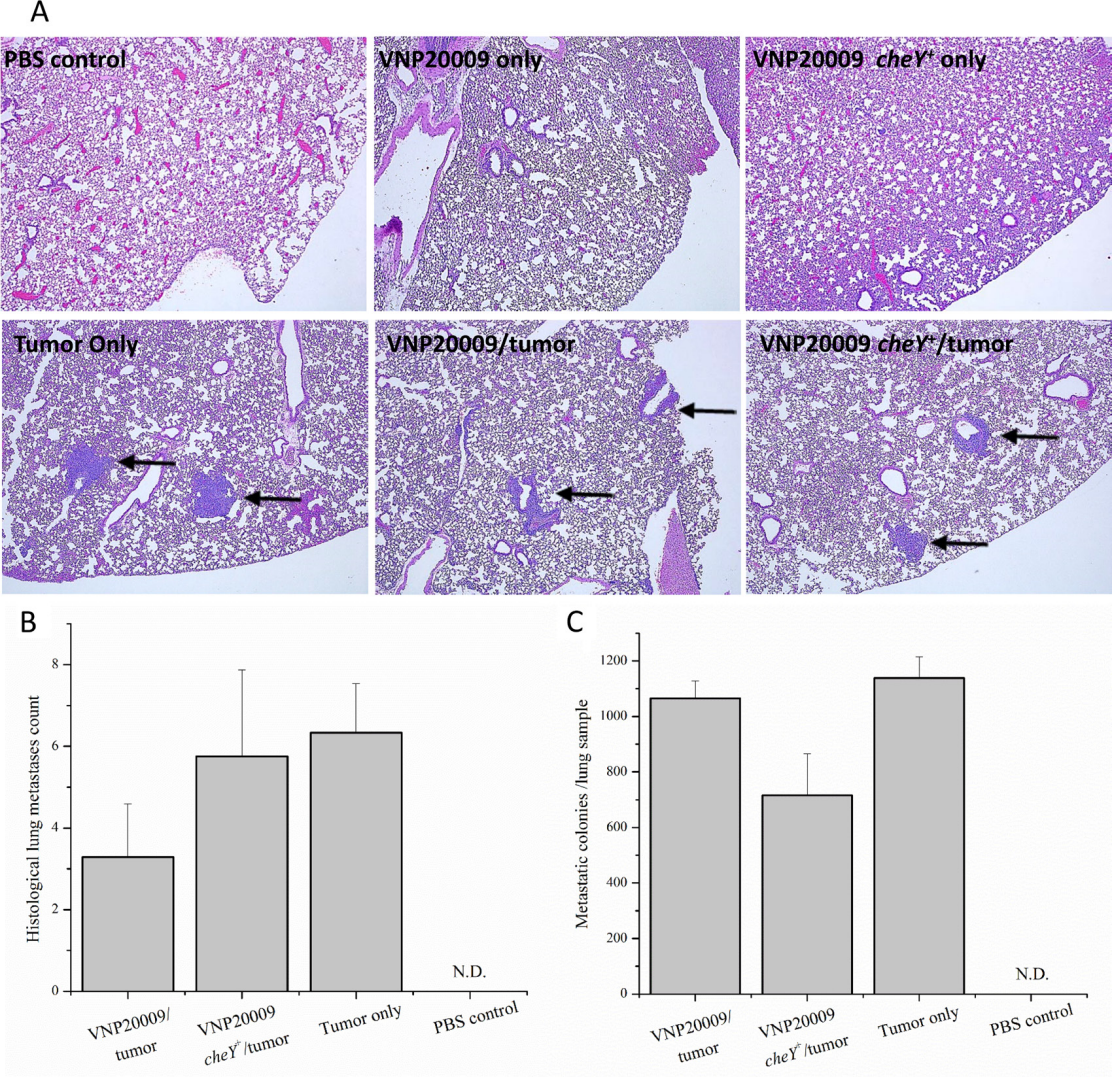
All 4T1-tumor bearing animals developed pulmonary metastases (**A**) Histologically, pulmonary metastases were identified as random accumulations of neoplastic epithelial cells often centered on small airways (arrow). Neither control animals nor animals injected with *S*. Typhimurium alone developed histologic evidence of metastasis. (**B**) The large lung lobe from each animal was placed in formalin and processed for H&E staining. The number of individual pulmonary metastases were counted per histologic section. (**C**) The small lung lobes from each animal were taken at necropsy and subsequently enzymatically digested. Cell suspensions were plated on cell culture plates and grown in media containing 6-thioguanine. After 8 days, metastatic colonies were fixed in methanol, stained with 0.03% methylene blue, and counted. PBS control, *n* = 3; VNP20009 only, *n* = 4; Tumor only, *n* = 3; VNP20009 and tumor, *n* = 7*;* VNP20009 *cheY*^+^ and tumor*, n* = 8. Error bars denote SEM, N.D, not detected.

Systemic metastasis is an important cause of death in women with breast cancer. Previous publications proposed that the use of attenuated, tumor-targeting *S*. Typhimurium strains can decrease metastatic tumorigenesis in mice without having systemic effects [32, 33]. The 4T1 model is not only a well-established and commonly used model for primary mammary tumorigenesis, but also for systemic metastasis [25]. In our experience, tumor cells reliably metastasize to the lungs by the end of week 2 following 4T1 cell injection (data not shown). To investigate this phenomenon further, we evaluated pulmonary metastasis using both qualitative and quantitative methodologies. At necropsy, the large lung lobe was removed and prepared for histopathology evaluation by a board certified veterinary pathologist (S.C.O.). Individual tumor cell aggregates (classified as metastases) were identified and counted. Histopathological analysis of pulmonary metastasis revealed no differences between the number of tumor cell aggregates identified histologically among any of the tumor-bearing groups, regardless of exposure to either *S*. Typhimurium strain (Figure 3A and 3B). 4T1 cells are unique in their inherent resistance to 6-thioguanine, a purine agonist that is lethal to most cells. Therefore, 4T1 metastatic cells can be grown in media supplemented with 6-thioguanine until they form small colonies, which enables a quantitative evaluation of lung metastasis. Concurrent with our histopathologic evaluation, our results confirm that no significant differences in numbers of metastatic colonies were identified among any of the tumor-bearing groups (Figure 3C). We attempted to discern the presence of *Salmonella* in fixed lung samples by reverse transcription followed by 16S RNA PCR amplification and by immunohistochemistry. However, we were unsuccessful in verifying the presence of VNP20009 in our lung tissue specimens using these approaches. It is possible that we did not detect the *Salmonella* due to technical limitations of our assay (low amounts or poor quality of RNA) or the quantity of *Salmonella* in the lungs were below the level of detection for our methodology.

### All animals bearing tumors and infected with *S*. Typhimurium developed significant liver disease

**Figure 4:**
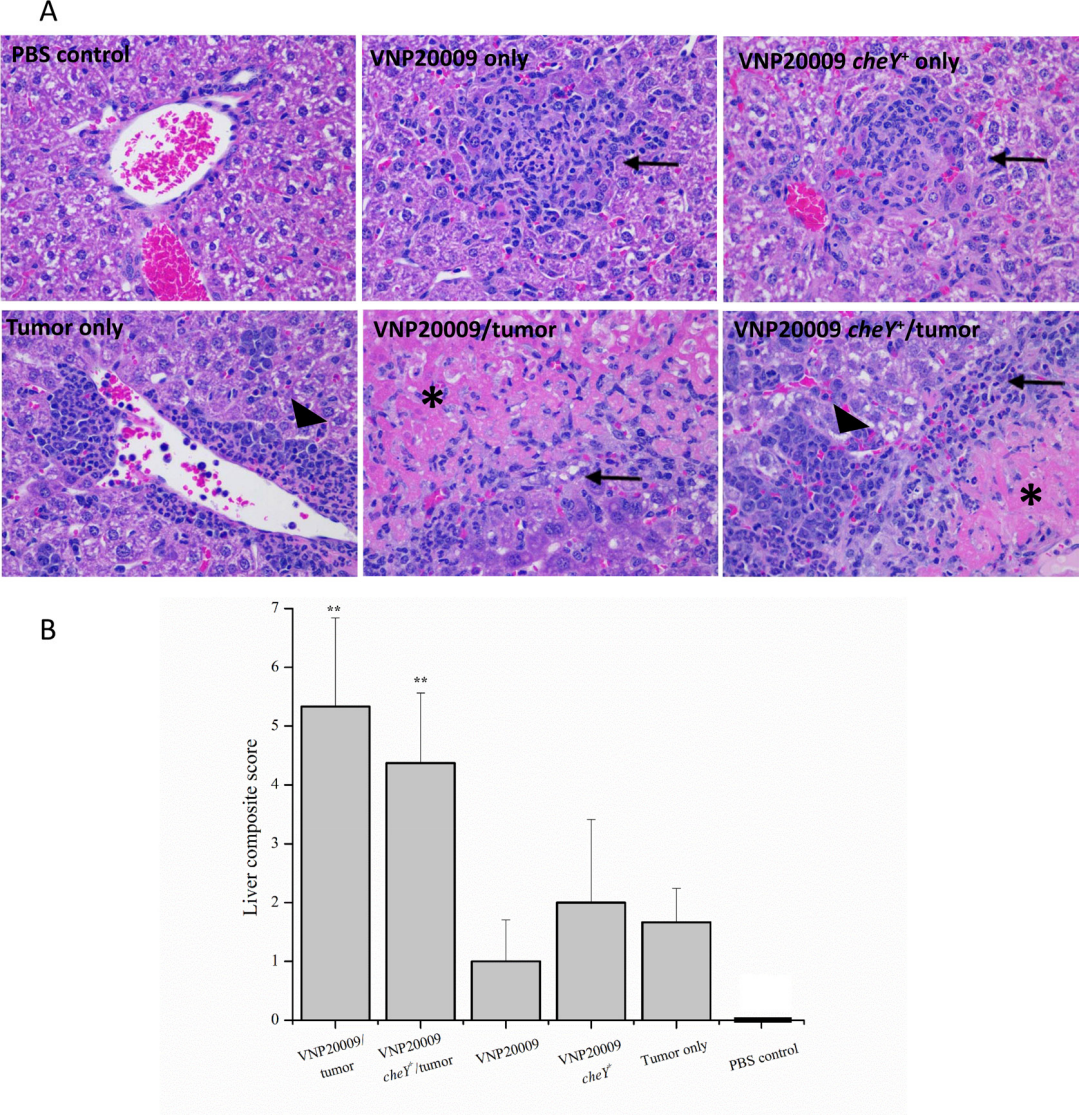
All experimental animals developed liver lesions (**A**) Animals receiving intravenous *S*. Typhimurium developed mild to moderate, multifocal inflammation characterized by random aggregates of mononuclear cells and neutrophils (arrows). Animals receiving 4T1 mammary carcinoma cells developed large amounts of extramedullary hematopoiesis predominantly in the area of large and small vessels, but also occasionally scattered throughout the parenchyma (large arrowheads). These areas were characterized by a heterogeneous group of erythroid and myeloid precursors as well as megakaryocytes. Those mice receiving both *S*. Typhimurium (regardless of strain) and 4T1 cells had significant amounts of inflammation (arrow) and extramedullary hematopoiesis (large arrowhead), as well as, large foci of liver necrosis (asterisk). (**B**) Inflammation, extramedullary hematopoiesis, and necrosis were individually scored for each sample. Individual scores were then summed to create a composite liver histopathology score for each animal. PBS control, *n* = 3; VNP20009 only, *n* = 2; VNP20009 *cheY+* only, *n* = 2; 4T1 only, *n* = 3; VNP20009 and tumor, *n* = 6*;* VNP20009 *cheY*^+^*, n* = 8. Error bars denote SD. Statistical significance was determined in relation to the tumor only sample by a two-tailed Student's *T-test* (**p < 0.01).

A very important finding in this study was the presence of significant morbidity in tumor-bearing animals exposed to either VNP20009 strain (Figure 1). *S*. Typhimurium VNP20009 has not previously been associated with significant morbidity in animal models, but instead is considered a safe strain to evaluate the effects of bacterial colonization on tumor development [34, 35]. Additionally, we found it interesting that the morbidity seen in tumor-bearing animals exposed to *S*. Typhimurium was not also seen in the non-tumor bearing animals exposed to the same bacteria (Figure 1). The increased morbidity cannot be explained by differences in primary tumor size or numbers of pulmonary metastases, because these were not significantly different from 4T1 tumor-bearing animals not exposed to *S*. Typhimurium (Figures 2 and 3). To evaluate the potential systemic effects of *S*. Typhimurium, livers of all animals were examined grossly and histologically. At necropsy, tumor-bearing animals infected with *S*. Typhimurium had gross evidence of liver damage. Livers from this animal group had large, sharply demarcated foci that were pale yellow and most often present at the edges of liver lobes (data not shown). No gross abnormalities were identified in the livers of any other treatment or control groups. Liver samples from all animals were processed for histopathology. These were then evaluated and scored by a board certified veterinary pathologist (S.C.O.) for amounts of inflammation, extramedullary hematopoiesis (EMH), and necrosis. Histopathological assessments revealed that liver lesions were significantly different in each of the treatment groups (Figure 4A). Livers from control animals were characterized by no or rare, small aggregates of mononuclear cells that made up less than 1% of the section (Figure 4A). Livers from animals only infected with either strain of *S*. Typhimurium were characterized by randomly scattered, variably-sized foci of inflammation composed of aggregates of mononuclear cells often admixed with neutrophils (Figure 4A). Livers from animals bearing mammary tumors only (with no exposure to *S*. Typhimurium) were characterized by multiple, variably-sized foci of EMH composed of aggregates of both immature myeloid and erythroid progenitors (Figure 4A). These aggregates were most commonly isolated to portal tracts; however, aggregates could be also identified randomly scattered throughout the parenchyma. Livers from tumor-bearing animals infected with either *S*. Typhimurium strain had large areas of inflammation similar to the bacteria-only treated groups and large amounts of EMH similar to 4T1-only animals (Figure 4A). However, these mice additionally displayed large coalescing foci of necrosis characterized by dead hepatocytes and replacement by fibrin (Figure 4A). To more quantitatively evaluate these histologic changes, scores were generated to reflect levels of inflammation, EMH, and necrosis. These individual scores were then totaled to generate a composite score for each animal (Figure 4B). All animals receiving any experimental treatment exhibited composite liver scores > 1 while control animals exhibited composite liver scores equal to 0. Results revealed that the composite liver scores from those tumor-bearing animals exposed to either strain of VNP20009 were significantly higher than any other treatment group (Figure 4B). These results suggested that the presence of two types of disease may lead to a more complicated systemic health status than either disease alone.

### *S*. Typhimurium chemotaxis did not contribute to mammary tumor colonization

**Figure 5:**
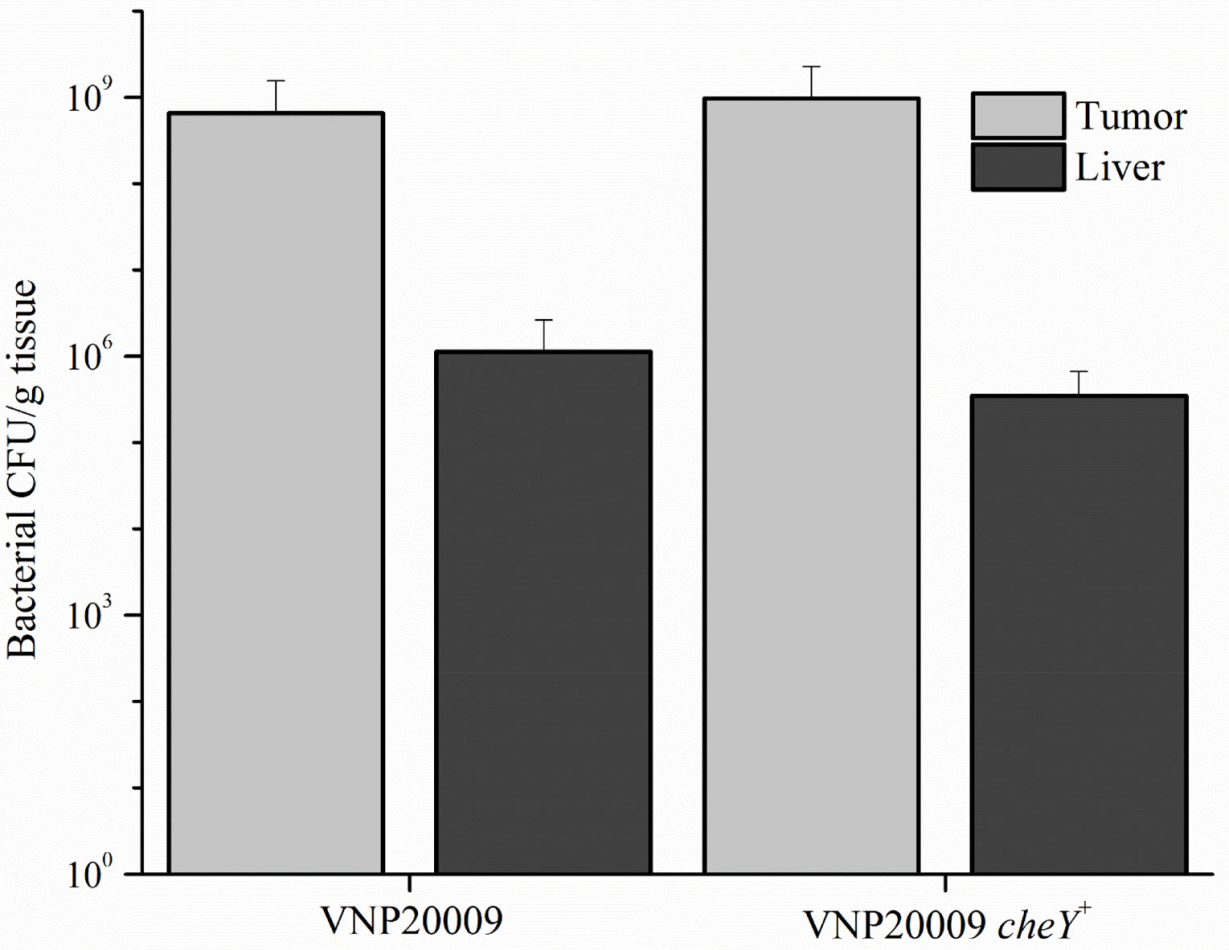
Bacterial colonization of tumor and liver tissue Intravenous injected VNP20009 or VNP20009 *cheY*^+^ were respectively recovered from tumor and liver samples and enumerated. No statistical differences were found between any groups. Data represents VNP20009, *n* = 6; VNP20009 *cheY*^+^, *n* = 8, with error bars denoting SD.

The efficiency of bacterial tumor-targeting was evaluated by the ratio of tumor to liver colonization. Tissue homogenates were serially diluted and dilutions were plated on nutrient agar to obtain bacterial colony counts. No differences were present upon comparing liver colonization of mice that did not receive 4T1 cells, but were only infected with *S*. Typhimurium (data not shown). Primary tumor and liver colonization by VNP20009 and VNP20009 *cheY*^+^, respectively, were not significantly different (Figure 5). We observed a significant increase in numbers for both bacterial strains in tissues, with tumor:liver colonization ratios ranging from 675:1 and 2,800:1 for mice exposed to VNP20009 or VNP20009 *cheY*^+^, respectively.

### Route of *S*. Typhimurium injection did not significantly affect disease pathobiology

**Figure 6:**
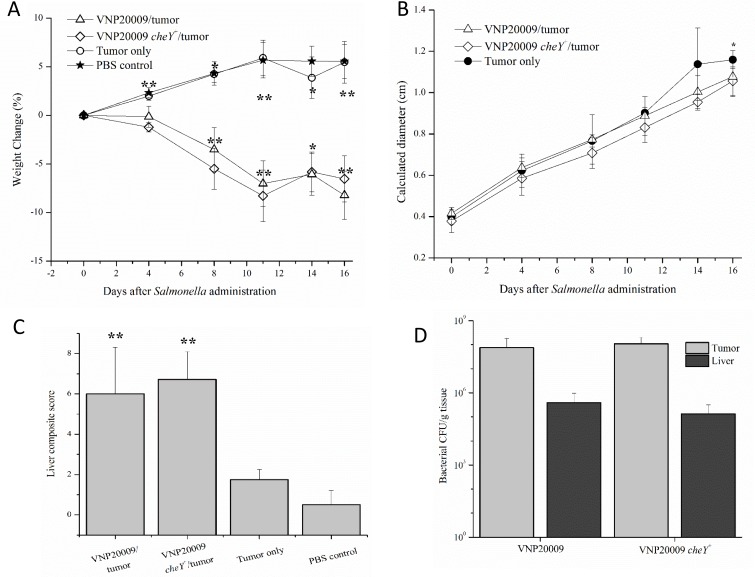
Disease pathobiology was evaluated following intratumoral injection of *S*. Typhimurium Six days following 4T1 tumor cell injections into the mammary fat pad, animals were administered either VNP20009 or VNP20009 *cheY*^+^ directly into mammary tumors. Similar parameters were assessed as in the previous intravenous experiment including (**A**) weight change, (**B**) calculated diameter of the primary mammary tumor, (**C**) histopathologic scoring of liver sections, and (**D**) number of bacterial cells recovered from the liver or primary tumor of infected animals. PBS control, *n* = 2; Tumor only, *n* = 4; VNP20009 and tumor*, n* = 6*;* VNP20009 *cheY*^+^ and tumor*, n* = 7. Error bars denote SEM for A and B, SD for C and D. Statistical significance was determined in relation to the tumor only sample by a two-tailed Student's *T-test* (*p < 0.05, **p < 0.01).

To investigate whether the delivery route of *S*. Typhimurium to the animal could influence its effects on tumor growth and disease pathobiology, experiments were repeated following direct injection of the bacterial strains into the tumor. Tumors were allowed to develop and progress for 6 days before VNP20009 or VNP20009 *cheY*^+^ (or PBS for control animals) were injected directly into the tumor at a concentration of approximately 2 × 10^4^ CFU/mouse. Similar to the previous experiment, tumor-bearing animals injected with either VNP20009 strain displayed decreased weight gain when compared to the remaining groups (Figure 6A). No clinically relevant differences were present in the calculated diameter of the mammary tumors in tumor-bearing animals independent of *S*. Typhimurium presence (Figure 6B). Tumor-bearing mice infected with either strain of VNP20009 presented a higher liver composite score than tumor only bearing mice or the PBS-control mice (Figure 6C). Primary tumor and liver colonization by VNP20009 and VNP20009 *cheY*^+^ did not differ significantly (Figure 6D). However, both bacterial strains were retained in the tumor, with tumor:liver colonization ratios ranging from 195:1 and 820:1 for mice exposed to VNP20009 or VNP20009 *cheY*^+^, respectively.

## DISCUSSION

We have presented a detailed report on the status of mice bearing 4T1 mammary carcinoma treated with *S*. typhimurium VNP20009. Historically, this strain of *Salmonella* has been attributed as a safe anticancer agent in the context of several murine tumor models [34, 35]. These studies culminated in the 2001 phase 1 human clinical trial using VNP20009 for the treatment of metastatic melanoma which ultimately found that though VNP20009 appeared to be safe for the use in human patients, its anti-tumor effects were equivocal [17]. Based on this, we formulated an experimental design to test both the safety and efficacy of *S*. Typhimurium as a treatment in the 4T1 mammary carcinoma model, as well as, the potential contribution of bacterial chemotaxis to primary tumor localization and metastatic potential to the lungs. Gaining a better understanding of how the bacteria react in different tumor microenvironments will be essential to future uses of VNP20009 as a cancer bio-therapeutic agent.

The majority of research analyzing the use of *Salmonella* as an anti-cancer agent has focused on its primary tumor burden effects. Fewer studies have been undertaken evaluating its ability to attenuate metastatic tumor burden. The 4T1 mammary carcinoma model represents a more natural study of metastatic tumors, as it accounts for the heterogeneity of cells in a primary tumor and allows for the biological selective pressures encountered by tumor cells to drive metastatic potential. This is opposed to artificial metastasis models that are based on the direct intravenous injection, where all tumor cells are placed into circulation to settle and grow into new tumors [36]. Perhaps the most relevant data pertinent to the present study evaluated *S*. Typhimurium A1-R in the 4T1 model using nude mice [36]. Here, the authors orthotopically-injected mice and surgically resected the primary tumor once it was established to maximize evaluation of brain metastasis [36]. In this particular model, *S*. Typhimurium A1-R was able to arrest the growth of breast-cancer associated brain metastasis and increased the survival of the orthotopically-transplanted, primary tumor resected mice [36]. In our present study, we chose to focus on lung metastasis, as the lung is the most common metastasis site of breast cancer found at autopsy and lung metastasis is also a prominent feature of the 4T1 model [25, 37]. While we observed a trending decrease in lung metastases in one of the quantitative assessments for the VNP20009 *cheY*^+^ strain (Figure 3C), the consensus data revealed only a minor reduction in metastatic burden (Figure 3). There are a variety of suggested reasons for the discrepancies found between the A1-R and VNP20009 studies, including the *Salmonella* strains utilized, the site of metastasis evaluated, the aggressiveness of the tumor model (surgically resected primary tumor versus non-resected), and the immune status of the animals. Together, these data emphasize the need to not only better define the genetic and phenotypical differences of *S*. Typhimurium strains in the context of different cancer sub-types, but also in the context of metastasis.

Beyond the ability of attenuated *Salmonella* strains to combat various types of cancer, assessments associated with the safety of the bacterial strain for use as a bio-therapeutic agent is also paramount. Many prior studies have been conducted evaluating the safety of VNP20009 and this particular strain likely has the best characterized safety profile of any of the therapeutic *Salmonella* strains. However, even though this strain of *Salmonella* is considered attenuated, there are significant clinical side effects related to its use [17, 38]. Indeed, one of the most striking aspects of our study is the observation that all tumor bearing animals exposed to VNP20009 exhibited significant morbidity associated with severe liver disease, which was not observed in any other experimental group of animals (Figure 4). One possible explanation for the increased morbidity and liver disease in tumor-bearing mice exposed to bacteria is that the mammary tumor could be acting as a reservoir for bacterial infection. We were unable to identify evidence of significant differences in bacterial burdens in tumor tissues taken at necropsy via immunohistochemistry. Moreover, no significant differences were identified in bacterial CFU identified in the livers from tumor-bearing versus non tumor-bearing mice (data not shown) suggesting that the systemic bacterial burdens were similar between the two groups. A second explanation for the observed morbidity and liver disease is that 4T1 mammary carcinoma cells have been previously reported to secrete soluble factors inducing immunosuppression [39]. Thus, it is possible that tumor-bearing animals exposed to bacteria were unable to control their systemic bacterial infections as well as those animals that did not have tumors and exposed to bacteria.

While some prior studies have reported increased morbidity and liver pathology accompanied by VNP20009 treatment, this particular side effect has not been extensively characterized and the significance of these liver outcomes has typically been underestimated [40, 41]. A previous study evaluating the administration of VNP20009 using the B16F10 tumor model, found that bacteria treatment mildly attenuated tumor growth and reported the presence of small foci of neutrophilic inflammation throughout the liver that increased over time and occurrence of hepatocyte necrosis [40]. However, this pathology was presented as mild and the liver appeared to recover over the course of the model [40]. Similarly, in another study evaluating the toxicity of VNP20009 and A1-R strains of *Salmonella* in a Lewis lung carcinoma model in nude mice, non-tumor bearing animals were intravenously administered two different doses of each bacterial strain [41]. By day 3, hemorrhagic foci were identified in the livers of all mice injected with bacteria, but no evaluation of liver histopathology was performed in tumor-bearing mice exposed to *Salmonella* [41]. Consistent with these prior studies, we also observed relatively mild liver lesions following VNP20009 treatment when administered alone (Figure 4). However, in the 4T1 mammary carcinoma model, we detected large areas of EMH in the liver, which was not reported in the other cancer models (Figure 4). Likewise, the combination of 4T1 mammary carcinoma and VNP20009 treatment appeared to act synergistically, causing significantly increased inflammation, EMH, and liver necrosis that ultimately resulted in increased morbidity (Figure 4). Thus, despite the clinical potential of VNP20009, more data associated with the safety of this bacterial strain is clearly necessary.

Despite the absence of anticancer effects by the *S*. Typhimurium strains tested, bacteria were retained in the tumor after 8 days, with an average tumor to liver colonization of 580:1 and 2,800:1 by VNP20009 and VNP20009 *cheY*^+^, respectively (Figure 5). For a variety of *in vivo* tumor models and timelines, including murine melanoma after 3 days and human melanoma and human colon carcinoma after 5 days, VNP20009 has been reported to have a tumor to liver colonization ratio at ≥ 1,000:1 which is in line with our data [12]. The targeting of *Salmonella* to tumor tissue remains a significant barrier in therapeutic applications (reviewed in [20]). Any and all improvements in this regard are critical. Our data reveals that chemotaxis does not significantly contribute to *S*. Typhimurium VNP20009 colonization of the primary 4T1 mammary tumor (Figure 5). The nature in which VNP20009 was constructed, by UV and chemical mutagenesis, left the strain with several genetic alterations, including 50 non-synonymous SNPs and the loss of 128 genes in the Suwwan deletion region [42]. Since restoration of one of the genes containing a non-synonymous SNP, *cheY*, did not significantly facilitate the promotion of tumor colonization by VNP20009, this raises the question if the remaining alterations in the genome are assisting or hindering the strain from its full tumor targeting potential.

To evaluate the possibility that the route of administration could have negatively influenced *S*. Typhimurium VNP20009 effectiveness in the 4T1 breast cancer model, we evaluated both intravenous and intratumoral injection. We originally speculated the following: (i) a direct injection into the tumor would be less likely to be associated with negative systemic affects, including liver lesions; and (ii) direct injection of a chemotaxis null strain would increase tumor colonization compared to the same strain systemically administered. However, we observed similar results between the two injection models. In both cases, tumor-bearing animals exposed to either VNP20009 strain had increased morbidity due to a high frequency of severe liver lesions not identified in the other treatment groups and no significant differences in tumor colonization regardless of the route of administration. In sum, we were unable to identify any significant differences in disease pathobiology between intravenous and intratumoral injection.

The data presented here expands the growing body of literature that suggests that individual *S*. Typhimurium strains with unique phenotypical characteristics will have differing levels of success as a cancer bio-therapeutic agent, depending on the cancer sub-type being targeted and the specific tumor microenvironmental niches present in the model. Additional studies are both necessary and ongoing to determine the safest and most efficacious utilization of *S*. Typhimurium VNP20009 in future anticancer therapies.

## MATERIALS AND METHODS

### Experimental animals

All experiments were conducted under institutional IACUC approval and in accordance with the NIH Guide for the Care and Use of Laboratory Animals. All experiments were conducted with 6–10 week-old, female BALB/C mice purchased from Jackson Laboratories. Animals were allowed to acclimate in the facilities for one week prior to tumor injections.

### Cell culture and injection

4T1 cells were grown under standard cell culture conditions in Roswell Park Memorial Institute (RPMI 1640) supplemented with 10% fetal bovine serum (FBS), 100 units/ml penicillin, and 100 μg/ml streptomycin. Cells used for injection were grown for 4–6 generations, washed, and re-suspended in sterile phosphate-buffered saline (PBS) prior to injection. Mice were anesthetized and maintained on isoflurane anesthesia throughout the tumor injection procedure. 1.2 × 10^6^ cells were injected into the mammary fat pad of each mouse. Control mice received the same volume of sterile PBS. Mice were recovered on room air. Mice were monitored 3 times per week, and weights and tumor measurements were recorded twice weekly. Calipers were used to measure two perpendicular diameters of each tumor. These were used to then determine a calculated tumor diameter [25]. Animals were euthanized when (i) weight loss exceeded 10–15% of original body weight, (ii) tumor growth reached 1.4–1.6 cm of calculated diameter, or (iii) if considered clinically moribund. In our experience with this model, measurable subcutaneous mammary tumors develop within 2–4 days of injection. These tumors progress reliably with evidence of metastatic disease present in the lungs by week 2 (unpublished data). By the end of 30 days, the tumors are approximately 1.4–1.6 cm in calculated diameter.

### Bacterial strains, growth and injection conditions

*S*. Typhimurium VNP20009 and VNP20009 *cheY*^+^ were grown in MSB media (1% tryptone, 0.5% yeast extract, 2 mM MgSO_4_, 2 mM CaCl_2_) at 37°C to an OD_600_ of 1.0, washed 3 times with PBS and adjusted to the final concentration of 2 × 10^5^ CFU/mL. A 100 μL dose of this final concentration was injected i.v. via the tail vein at day 16 post tumor-injection, or directly into the tumor at day 6 post tumor-injection. Control mice were injected with an equal amount of PBS.

### Tissue collection and processing

At euthanasia, whole blood was collected via cardiac puncture and a full necropsy was performed. The large lung lobes were partially inflated in formalin and, along with samples of primary tumor and liver, fixed in formalin for at least 24 hours (h). They were then submitted for histopathology. Individual liver and primary tumor specimens were also collected for bacterial enumeration and storage at −80°C for downstream protein and nucleic acid extraction. For bacterial enumeration, specimens were weighed, homogenized using a hand held pestle system, serially diluted in PBS, plated on MSB plates, and incubated for 14 h at 37°C. The remaining smaller lung lobes were enzymatically digested using Type IV collagenase (collagenase from *Clostridium histolyticum*, Sigma-Aldrich) and porcine elastase (elastase from porcine pancreas, MPBiomedicals). After a series of washes in Hank's buffered saline solution (HBSS), cells were resuspended in Dulbecco's modified eagle medium (DMEM) with 10% FBS, 100 units/ml penicillin, and 100 μg/ml streptomycin, 60 μM 6-thioguanine, and plated onto cell culture plates. After 5–10 days of incubation at 37°C and 5% CO_2_, media was removed, cells were briefly fixed in methanol, stained with 0.03% new methylene blue, and tumor colonies were counted (Supplementary Figure 1).

### Histopathology

Formalin-fixed tissues were embedded in paraffin and stained with routine hematoxylin and eosin (H&E) staining for histopathologic examination by a board certified veterinary pathologist (S.C.O). For lung sections, numbers of individual pulmonary tumors were counted in a single, 5 μm section. For each animal, the entire right lung lobe was embedded and sectioned at the level of the mainstem bronchus. For liver sections, a single, 5 μm section was evaluated. Amounts of inflammation, EMH, and necrosis were scored individually as follows: 0; no inflammation/EMH/necrosis identified, 1; 1% to 33% of the examined section was composed of inflammation/EMH/necrosis, 2; 34%–66% of the examined section was composed of inflammation/EMH/necrosis, and 3; greater than 67% of the section was composed of inflammation/EMH/necrosis. These individual scores were then summed to give a total composite score for each animal.

## SUPPLEMENTARY MATERIALS FIGURES



## References

[1] McCarthy EF, The toxins of William B (2006). Coley and the treatment of bone and soft-tissue sarcomas. Iowa Orthop J.

[2] Nauts HC, Swift WE, Coley BL (1946). The treatment of malignant tumors by bacterial toxins as developed by the late William B. Coley MD, reviewed in the light of modern research. Cancer Res.

[3] Wiemann B, Starnes CO (1994). Coley’s toxins, tumor necrosis factor and cancer research: a historical perspective. Pharmacol Ther.

[4] Rihova B, Stastny M (2015). [History of Immuno-therapy - from Coley Toxins to Check-points of the Immune Reaction]. Klin Onkol.

[5] Yazawa K, Fujimori M, Amano J, Kano Y, Taniguchi S (2000). Bifidobacterium longum as a delivery system for cancer gene therapy: selective localization and growth in hypoxic tumors. Cancer Gene Ther.

[6] Taniguchi S, Fujimori M, Sasaki T, Tsutsui H, Shimatani Y, Seki K, Amano J (2010). Targeting solid tumors with non-pathogenic obligate anaerobic bacteria. Cancer Sci.

[7] Theys J, Pennington O, Dubois L, Anlezark G, Vaughan T, Mengesha A, Landuyt W, Anne J, Burke PJ, Durre P, Wouters BG, Minton NP, Lambin P (2006). Repeated cycles of Clostridium-directed enzyme prodrug therapy result in sustained antitumour effects in vivo. Br J Cancer.

[8] Silva-Valenzuela CA, Desai PT, Molina-Quiroz RC, Pezoa D, Zhang Y, Porwollik S, Zhao M, Hoffman RM, Contreras I, Santiviago CA, McClelland M (2016). Solid tumors provide niche-specific conditions that lead to preferential growth of Salmonella. Oncotarget.

[9] Leschner S, Weiss S (2010). Salmonella-allies in the fight against cancer. J Mol Med (Berl).

[10] Nallar SC, Xu DQ, Kalvakolanu DV (2016). Bacteria and genetically modified bacteria as cancer therapeutics: Current advances and challenges. Cytokine.

[11] Pawelek JM, Low KB, Bermudes D (1997). Tumor-targeted Salmonella as a novel anticancer vector. Cancer Res.

[12] Clairmont C, Lee KC, Pike J, Ittensohn M, Low KB, Pawelek J, Bermudes D, Brecher SM, Margitich D, Turnier J, Li Z, Luo X, King I (2000). Biodistribution and genetic stability of the novel antitumor agent VNP20009, a genetically modified strain of Salmonella typhimurium. J Infect Dis.

[13] Zhao M, Yang M, Ma H, Li X, Tan X, Li S, Yang Z, Hoffman RM (2006). Targeted therapy with a Salmonella typhimurium leucine-arginine auxotroph cures orthotopic human breast tumors in nude mice. Cancer Res.

[14] Eisenstark A, Kazmierczak RA, Dino A, Khreis R, Newman D, Schatten H (2007). Development of Salmonella strains as cancer therapy agents and testing in tumor cell lines. Methods Mol Biol.

[15] Wang CZ, Kazmierczak RA, Eisenstark A (2016). Strains, Mechanism, and Perspective: Salmonella-Based Cancer Therapy. Int J Microbiol.

[16] Low KB, Ittensohn M, Luo X, Zheng LM, King I, Pawelek JM, Bermudes D (2004). Construction of VNP20009: a novel, genetically stable antibiotic-sensitive strain of tumor-targeting Salmonella for parenteral administration in humans. Methods Mol Med.

[17] Toso JF, Gill VJ, Hwu P, Marincola FM, Restifo NP, Schwartzentruber DJ, Sherry RM, Topalian SL, Yang JC, Stock F, Freezer LJ, Morton KE, Seipp C (2002). Phase I study of the intravenous administration of attenuated Salmonella typhimurium to patients with metastatic melanoma. J Clin Oncol.

[18] Crull K, Bumann D, Weiss S (2011). Influence of infection route and virulence factors on colonization of solid tumors by Salmonella enterica serovar Typhimurium. FEMS Immunol Med Microbiol.

[19] Crull K, Rohde M, Westphal K, Loessner H, Wolf K, Felipe-Lopez A, Hensel M, Weiss S (2011). Biofilm formation by Salmonella enterica serovar Typhimurium colonizing solid tumours. Cell Microbiol.

[20] Chorobik P, Czaplicki D, Ossysek K, Bereta J (2013). Salmonella and cancer: from pathogens to therapeutics. Acta Biochim Pol.

[21] Kasinskas RW, Forbes NS (2006). Salmonella typhimurium specifically chemotax and proliferate in heterogeneous tumor tissue in vitro. Biotechnol Bioeng.

[22] Kasinskas RW, Forbes NS (2007). Salmonella typhimurium lacking ribose chemoreceptors localize in tumor quiescence and induce apoptosis. Cancer Res.

[23] Stritzker J, Weibel S, Seubert C, Gotz A, Tresch A, van Rooijen N, Oelschlaeger TA, Hill PJ, Gentschev I, Szalay AA (2010). Enterobacterial tumor colonization in mice depends on bacterial metabolism and macrophages but is independent of chemotaxis and motility. Int J Med Microbiol.

[24] Broadway KM, Denson EA, Jensen RV, Scharf BE (2015). Rescuing chemotaxis of the anticancer agent Salmonella enterica serovar Typhimurium VNP20009. J Biotechnol.

[25] Pulaski BA, Ostrand-Rosenberg S (2001). Mouse 4T1 breast tumor model. Curr Protoc Immunol.

[26] Zhao M, Yang M, Li XM, Jiang P, Baranov E, Li S, Xu M, Penman S, Hoffman RM (2005). Tumor-targeting bacterial therapy with amino acid auxotrophs of GFP-expressing Salmonella typhimurium. Proc Natl Acad Sci USA.

[27] Zhao M, Geller J, Ma H, Yang M, Penman S, Hoffman RM (2007). Monotherapy with a tumor-targeting mutant of Salmonella typhimurium cures orthotopic metastatic mouse models of human prostate cancer. Proc Natl Acad Sci USA.

[28] Nagakura C, Hayashi K, Zhao M, Yamauchi K, Yamamoto N, Tsuchiya H, Tomita K, Bouvet M, Hoffman RM (2009). Efficacy of a genetically-modified Salmonella typhimurium in an orthotopic human pancreatic cancer in nude mice. Anticancer Res.

[29] Hayashi K, Zhao M, Yamauchi K, Yamamoto N, Tsuchiya H, Tomita K, Hoffman RM (2009). Cancer metastasis directly eradicated by targeted therapy with a modified Salmonella typhimurium. J Cell Biochem.

[30] Kimura H, Zhang L, Zhao M, Hayashi K, Tsuchiya H, Tomita K, Bouvet M, Wessels J, Hoffman RM (2010). Targeted therapy of spinal cord glioma with a genetically modified Salmonella typhimurium. Cell Prolif.

[31] Lee KC, Zheng L, Luo X, King I (2000). Comparative Evaluation of the Acute Toxic Effects in Monkeys, Pigs and Mice of a Genetically Engineered Salmonella Strain (VNP20009) Being Developed as an Antitumor Agent. International Journal of Toxicology.

[32] Miwa S, Yano S, Zhang Y, Matsumoto Y, Uehara F, Yamamoto M, Hiroshima Y, Kimura H, Hayashi K, Yamamoto N, Bouvet M, Tsuchiya H, Hoffman RM (2014). Tumor-targeting Salmonella typhimurium A1-R prevents experimental human breast cancer bone metastasis in nude mice. Oncotarget.

[33] Matsumoto Y, Miwa S, Zhang Y, Hiroshima Y, Yano S, Uehara F, Yamamoto M, Toneri M, Bouvet M, Matsubara H, Hoffman RM, Zhao M (2014). Efficacy of tumor-targeting Salmonella typhimurium A1-R on nude mouse models of metastatic and disseminated human ovarian cancer. J Cell Biochem.

[34] Sznol M, Lin SL, Bermudes D, Zheng LM, King I (2000). Use of preferentially replicating bacteria for the treatment of cancer. J Clin Invest.

[35] Luo X, Li Z, Lin S, Le T, Ittensohn M, Bermudes D, Runyab JD, Shen SY, Chen J, King IC, Zheng LM (2001). Antitumor effect of VNP20009, an attenuated Salmonella, in murine tumor models. Oncol Res.

[36] Zhang Y, Miwa S, Zhang N, Hoffman RM, Zhao M (2015). Tumor-targeting Salmonella typhimurium A1-R arrests growth of breast-cancer brain metastasis. Oncotarget.

[37] Weigelt B, Peterse JL, van ‘t Veer LJ (2005). Breast cancer metastasis: markers and models. Nat Rev Cancer.

[38] Thamm DH, Kurzman ID, King I, Li Z, Sznol M, Dubielzig RR, Vail DM, MacEwen EG (2005). Systemic administration of an attenuated, tumor-targeting Salmonella typhimurium to dogs with spontaneous neoplasia: phase I evaluation. Clin Cancer Res.

[39] Kano A (2015). Tumor cell secretion of soluble factor(s) for specific immunosuppression. Sci Rep.

[40] Rosenberg SA, Spiess PJ, Kleiner DE (2002). Antitumor effects in mice of the intravenous injection of attenuated Salmonella typhimurium. J Immunother.

[41] Zhang Y, Zhang N, Zhao M, Hoffman RM (2015). Comparison of the selective targeting efficacy of Salmonella typhimurium A1-R and VNP20009 on the Lewis lung carcinoma in nude mice. Oncotarget.

[42] Broadway KM, Modise T, Jensen RV, Scharf BE (2014). Complete genome sequence of Salmonella enterica serovar Typhimurium VNP20009, a strain engineered for tumor targeting. J Biotechnol.

