# Supplementary material for: Effect of Salmonella enterica serovar Typhimurium VNP20009 and VNP20009 with restored chemotaxis on 4T1 mouse mammary carcinoma progression
Source: Oncotarget. 2017 Apr 4;8(20):33601–13. doi: 10.18632/oncotarget.16830 (PMC5464893; doi:10.18632/oncotarget.16830)
Supplement: Supplementary file 1 [file oncotarget-08-33601-s001.pdf]

Effect of *Salmonella enterica* serovar Typhimurium VNP20009 and VNP20009 with restored chemotaxis on 4T1 mouse mammary carcinoma progression

Supplementary Materials

A

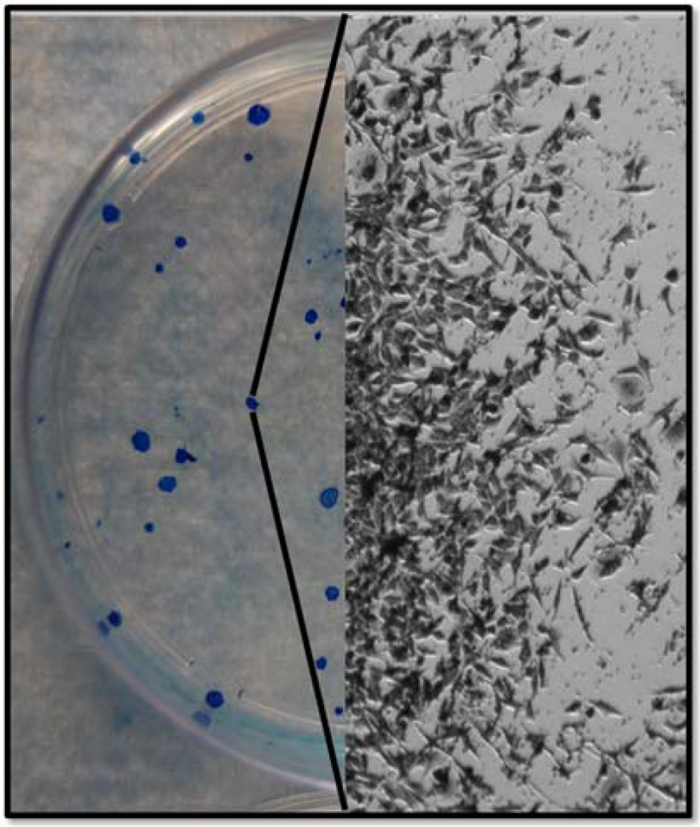

Supplementary Figure 1: Metastatic 4T1 cells can be isolated from organs of interest and grown in supplemented media to yield quantitative evaluations of metastatic tumor burden. Following necropsy, the small lung lobes were enzymatically digested, filtered, and grown on cell culture plates in media supplemented with 6-thioguanine. 4T1 cells are inherently resistant to 6-thioguanine and, thus, individual metastatic cells are able to grow into tumor cell colonies and manually counted.
